# Real-life experience in two cases of secondary prophylaxis with letermovir for CMV infection in solid organ transplantation

**DOI:** 10.1128/spectrum.01630-23

**Published:** 2023-10-30

**Authors:** Ana-Belén Pérez, Marta Santos Bravo, Elisa Vidal-Verdú, Aurora Páez-Vega, José-Manuel Vaquero-Barrios, José-Luis Montero, María-Ángeles Marcos, Julián Torre-Cisneros

**Affiliations:** 1 Microbiology Service, Reina Sofia University Hospital, Cordoba, Spain; 2 Maimónides Institute for Biomedical Research (IMIBIC), Cordoba, Spain; 3 Centre of Biomedical Research for Infectious Diseases (CIBERINFEC), Institute of Health Carlos III, Madrid, Spain; 4 Microbiology Service, Hospital Clínic, Barcelona, Spain; 5 Infectious Diseases Service, Reina Sofia University Hospital, Cordoba, Spain; 6 Maimónides Institute for Biomedical Research (IMIBIC), Cordoba, Spain; 7 Departament of Medicine (Medicine, Dermatology and Otorhinolaryngology), University of Cordoba, Spain; 8 Lung Transplantation Section, Reina Sofia University Hospital, Cordoba, Spain; 9 Liver Transplantation Section, Reina Sofia University Hospital, Cordoba, Spain; University of Brescia, Brescia, Italy

**Keywords:** cytomegalovirus, letermovir, secondary prophylaxis, transplant recipients, antiviral, resistance mutation

## Abstract

The use of valganciclovir as secondary prophylaxis for cytomegalovirus (CMV) infection in solid organ transplantation (SOT) is the most prevalent therapeutic choice, but it has been dismissed by the presence of resistance and toxicity. Letermovir (LMV) is indicated as primary prophylaxis in hematopoietic stem cell transplantation, but there is scarce clinical data on its use as secondary prophylaxis in SOT. We present two cases of SOT recipients (lung, liver) who underwent LMV secondary prophylaxis. One patient developed the L595S (*UL97*)-resistant mutation to ganciclovir/valganciclovir and experienced several CMV relapses under LMV therapy, but no LMV resistance mutations were detected. The second patient developed the C325F (*UL56*)-resistant mutation to LMV under secondary prophylaxis which was rescued by foscarnet. This observation reviews previously published clinical data on LMV secondary prophylaxis and recommends a cautious use of LMV in the clinical practice due to the early development of *UL56* resistance mutations.

**IMPORTANCE:**

This observation provides comprehensive data on the clinical correlates of both cytomegalovirus (CMV) genotypic follow-up and clinical monitoring and outcomes for two different solid organ transplantation recipients that received letermovir as secondary prophylaxis. Our study emphasizes that monitoring of CMV disease in the patient and early genotypic detection of resistance mutations are essential when using new antiviral drugs for off-label indication in patients experiencing CMV relapses or not responding to standard antiviral therapy. These cases and the bibliography reviewed can be helpful for other researchers and clinicians working in the field to optimize the use of new treatments for transplant recipients since drug-resistant CMV infection is an important emerging problem even with new developments in antiviral treatment.

## OBSERVATION

Clinical trials have demonstrated the efficacy and safety of letermovir (LMV) for primary prophylaxis of cytomegalovirus (CMV) infection in hematopoietic stem cell transplant (HSCT) patients (1) and in high-risk kidney transplant recipients (2). However, no trials have been conducted on the use of LMV as secondary prophylaxis for CMV disease. The drug of choice for this indication is valganciclovir (3, 4), which can cause resistance or neutropenia that may require discontinuation (3, 4). Foscarnet has many limitations that complicate its use, such as being nephrotoxic and needing to be administered intravenously (3, 4). The efficacy of maribavir has not been demonstrated in dose/response studies (5, 6). Therefore, LMV could be a potential therapeutic choice for this indication (1, 2).

### Case reports

**Fig 1:**
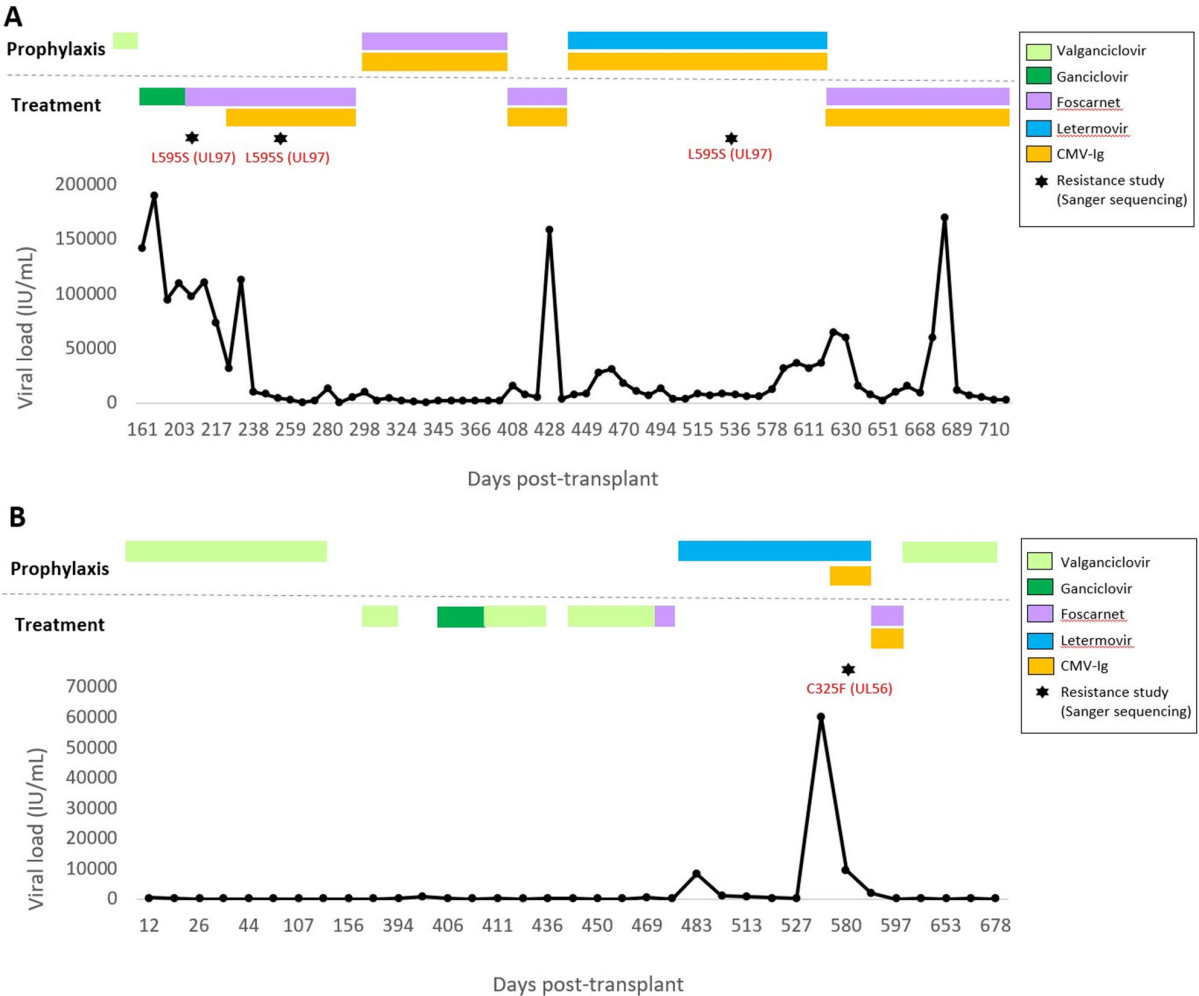
Description of the virologic course of the two clinical cases presented (panel A, lung transplant; panel B, liver transplant). CMV viral loads (IU/mL) were monitored over the time course after transplantation. Antiviral therapies are indicated according to prophylaxis or CMV treatment regimens. Resistance mutations detected by genotypic resistance studies are pointed with a star.

A 56-year-old CMV-seronegative female with idiopathic pulmonary fibrosis received a double lung transplant from a CMV-seronegative donor (D−/R−) in August 2018. Prophylaxis was initiated with valganciclovir, and 6 months after transplantation, the patient developed a breakthrough CMV primary infection (pneumonitis and plasma viral load of 142,000 IU/mL) (Fig. 1A). Subsequent treatment with ganciclovir failed, and a genotypic resistance study performed by Sanger sequencing showed the L595S mutation in the *UL97* gene (IC_50_ × 8.5–9.2; which confers a 8.5- to 9.2-fold decreased susceptibility to ganciclovir/valganciclovir) (7). Treatment with foscarnet combined with CMV-specific immunoglobulin and everolimus controlled the patient’s symptoms. After CMV infection was resolved, the patient started secondary prophylaxis with foscarnet and specific immunoglobulin weekly, which was maintained for approximately 5 months until nephrotoxicity forced the discontinuation of foscarnet. As a result, secondary prophylaxis with LMV (480 mg/24 h) was initiated. CMV load at the time of LMV secondary prophylaxis was 7,740 IU/mL. Prophylaxis was maintained for 6 months, and the patient remained asymptomatic although breakthrough replication was observed. Three months after starting treatment with LMV, a genotypic resistance study was performed and showed no mutations in the *UL56* gene (the plasma viral load was 7,800 IU/mL at this point). Only the L595S (*UL97*) mutation was detected over the clinical course of this patient. No mutations were found in *UL54* or *UL56* antiviral target genes. The patient developed chronic rejection that required increased immunosuppression. Six months after starting LMV, she developed CMV pneumonitis and plasma CMV viral load increased to 170,000 IU/mL; however, no genotypic resistance study was performed at this time. The patient died despite treatment with foscarnet combined with CMV-specific immunoglobulin.

A 39-year-old CMV-seronegative male with alcoholic liver cirrhosis received a liver transplant from a CMV-seropositive (D+/R−) donor in February 2021. The patient was administered primary prophylaxis with valganciclovir for 4.5 months, which was discontinued due to severe neutropenia (Fig. 1B). One year after transplantation, he developed gastrointestinal CMV disease (detected in biopsy) which was treated with valganciclovir until being discontinued due to neutropenia. The patient then developed CMV retinitis that required treatment with ganciclovir/valganciclovir. Fifteen months after transplant, he developed SARS-COV-2 pneumonia complicated by a pulmonary superinfection with *Pneumocystis jirovecii* and CMV. Valganciclovir was administered until being replaced with foscarnet due to neutropenia. After clinical and virological response (35 IU/mL), secondary prophylaxis with LMV (480 mg/24 h) was initiated. After 5 weeks of LMV administration, asymptomatic low-grade viral replication (229–1,230 IU/mL) was detected, but LMV was maintained for 4 months. Plasma CMV replication gradually increased until viral load reached 60,000 IU/mL. A genotypic resistance mutation study by Sanger sequencing showed the development of the C325F mutation (3,000-fold decrease susceptibility to LMV) in the *UL56* LMV target gene (8), and no resistance mutation was detected in either *UL97* or *UL54*. Although the patient was asymptomatic, LMV was discontinued and treatment with foscarnet combined with CMV-specific immunoglobulin was initiated. The patient is currently continuing secondary prophylaxis with valganciclovir.

### Clinical assessment

CMV treatment and monitoring were performed according to the clinical protocol of the center (Reina Sofia University Hospital, Cordoba).

### Viral load quantification

Quantification of CMV loads was performed by real-time polymerase chain reaction (qPCR) using the Cobas CMV kit on the Cobas 6800 System (Roche, Basel, Switzerland) according to the manufacturer’s instructions.

### Genotypic resistance study

Antiviral resistance studies were performed to detect resistant mutations in the antiviral target genes (*UL97, UL54, UL56*). This study was requested based on the virological response of the patient to the specific antiviral treatment received. Genotypic resistance studies were performed at the Reference Centre for antiviral resistance, Hospital Clinic of Barcelona, between April 2019 and September 2022. Nucleic acid extraction from plasma samples was performed using a MagNAPure Compact (Roche, Switzerland). Genotypic testing was done by Sanger sequencing based on PCR amplification of HCMV *UL97* (residues 270–670), *UL54* (300–1,000), and *UL56* (180–395) regions, followed by BigDye Terminator v3.1. (Applied Biosystems) dideoxy chain-termination sequencing using previously described primers and procedures (9 – 11). Sequences were analyzed and aligned using the MEGA v.7. software and were compared with the HCMV TB40 strain (GenBank: MF871618.1) using the MRA-Mutation Resistance Analyzer tool provided by the University of Ulm (12).

### Secondary prophylaxis with letermovir: review of the literature

Table 1 summarizes the published cases of secondary prophylaxis with LMV (13 – 17), as well as the two cases presented in this observation. A total of 13 transplant recipients have been reported (8 lung, 3 kidney, 1 heart, and 1 liver). All recipients were high-risk seronegative patients who received seropositive organs (D+/R−), except one who received a seronegative organ (D−/R−) that had a primary infection. All patients had previously received valganciclovir in addition to other antiviral therapies (foscarnet, cidofovir, leflunomide, CMV-specific immunoglobulin). Toxicity, resistance, or failure of other therapies leads to LMV administration as secondary prophylaxis. Seven patients developed CMV infection with confirmed viremia while on secondary prophylaxis with LMV. Aryal et al. (13) reported three cases with breakthrough viremia, but no resistance study was performed. In the rest of the reports, four patients developed confirmed resistance to LMV with *UL56* mutations.

**TABLE 1:** Review of use of letermovir as secondary prophylaxis for cytomegalovirus infection after solid organ transplantation (only references with disaggregated data)^*b*^

Reference	SOT	D/R serology	Previous treatment/prophylaxis	Reason for letermovir prophylaxis	Previous CMV infection	CMV infection on letermovir prophylaxis	Letermovir resistance	Treatment	Outcome
(13)	Lung	D+/R−	Valganciclovir	Leukopenia	Viremia	No	No	Not applicable	Alive
(13)	Lung	D+/R−	Valganciclovir	Leukopenia	Viremia	No	No	Not applicable	Alive
(13)	Lung	D+/R−	Valganciclovir	Leukopenia	Viremia	No	No	Not applicable	Alive
(13)	Lung	D+/R−	Valganciclovir	Leukopenia	Viremia	Viremia (1,910 IU/mL)^*a*^	Not performed	Not available	Alive (failure therapy)
(13)	Lung	D+/R−	Valganciclovir	Leukopenia	Viremia	Viremia (33,392 IU/mL) **^*a*^**	Not performed	Not available	Alive (failure therapy)
(13)	Lung	D+/R−	Valganciclovir	Resistance (UL97 mutation)	Viremia	Viremia (2,499 IU/mL) **^*a*^**	Not performed	Not available	Death (for CMV infection)
(14)	Kidney	D+/R−	Valganciclovir Foscarnet	Resistance (UL97 mutations, L595F, H520Q, and M460V and UL54 mutation, A987G	CMV syndrome CMV colitis?	Intermittent viremia up to 200 copies/mL **^*a*^**	UL56 mutation, C325Y; persistence of the UL97 mutation M460V; and a new UL54 mutation P522S	Foscarnet, CMV immunoglobulin, everolimus	Alive (with functional graft)
(14)	Kidney	D+/R−	Valganciclovir Foscarnet CMV immune globulin	Resistance (UL97 mutation, C607F)	Disseminated disease	Viremia (13,200 copies/mL) **^*a*^**	UL56 mutation, C325Y	Foscarnet	Death (PTLD)
(15)	Heart	D+/R−	Valganciclovir* Cidofovir, foscarnet** Leflunomide***	*Resistance (UL 97 mutation, C603W) **Intolerance ***Failure	CMV syndrome with viremia	No	No	Not applicable	Alive
(16)	Kidney	D+/R−	Valganciclovir* Cidofovir**	*Resistance (UL 97 mutation, C603W) **Intolerance	Gastrointestinal CMV disease with viremia	Low-grade asymptomatic viremia	No	Cidofovir	Alive (graft failure)
(17)	Lung	D+/R−	Valganciclovir CMV immune globulin Leflunomide	Resistance (UL 97 mutation, M460V and UL54 mutation, L516P)	CMV syndrome with viremia	Viremia (4.1 log_10_ copies/mL) **^*a*^**	UL56 mutation C325Y (IC_50_ x >8,000) UL 97 mutation, M460V	Foscarnet	Alive (with functional graft)
Case 1	Lung	D−/R−	Valganciclovir* Foscarnet** CMV immune globulin	*Resistance (UL97 mutation, L595S). **Intolerance (nephrotoxicity)	Pneumonitis	Pneumonitis	No (3 months of treatment) Unknown at failure (6 months of treatment)	Foscarnet + CMV-specific immunoglobulins	Death (chronic rejection + CMV disease)
Case 2	Liver	D+/R−	Valganciclovir* Foscarnet	*Intolerance (leukopenia)	CMV colitis	Viremia (60,000 IU/mL)	UL56 mutation, C325F (IC_50_ x >3.000)	Foscarnet + CMV-specific immunoglobulins	Alive

^*a*^ Viral loads are expressed in IU/mL or copies/mL according to the data reported in the cited studies.

^*b*^ CMV, cytomegalovirus; IC50, 50% inhibitory concentration; PTLD, post-transplant lymphoproliferative disorder.

It is difficult to review the literature because the descriptions include cases of both prolonged treatment and secondary prophylaxis. This is what occurs with the 27 cases with SOT reported by Linder et al. (18) and in the 16 cases described by Saullo et al. (19). In the report of Linder et al. (18), 30% of 27 recipients patients were treated for graft rejection in the 3 months prior to initiation of LMV, and the most frequent indications for initiating LMV were intolerance to other agents (77%) and detection of proven antiviral resistance to others antiviral (32%). This study included 9 patients with SOT who initiated LMV with a viral load >1,000 IU/mL, of whom 5 had a reduction in viral load of at least 1 log 2–4 weeks after initiating LMV and 3/9 patients had a viral load <1,000 IU/mL at the time of stopping LMV treatment. One of the patients who had a CMV viral load rebound at week 9–12 post-LMV treatment had the C325Y mutation in the *UL56*. Two other patients died. In the cases with SOT published by Saullo et al. (19), valganciclovir was discontinued in 10 cases due to myelosuppression, mainly leukopenia, and in 6 cases due to resistance mutations in both *UL97* and *UL54* genes. One patient developed breakthrough viremia that responded to foscarnet, and a resistance study for the *UL56* gene was not performed. Ten patients developed low-grade viremia that did not require treatment (no resistance studies were performed).

In conclusion, focusing on the 27 cases using LMV as secondary prophylaxis from the articles reviewed (13 – 17, 19), LMV was successful in seven cases, it failed in eight cases that displayed increasing CMV viremia, and LMV prophylaxis was discontinued in three other cases for other reasons (lack of insurance coverage, transition to palliative care, or loss to follow-up due to transfer to another medical center); therefore, control of viral replication was achieved in 46.7% (7/15) of the cases. The remaining nine cases were still on prophylaxis at the time of study completion.

In 12 of the 27 cases, resistance mutations were detected in *UL97* and *UL54*, so it was decided that LMV would be used as a prophylactic option. Of the eight cases in which viremia could not be controlled with LMV, resistance in *UL56* was assessed in four patients, and mutations were detected in three of them (75%).

### Discussion

The main reasons of valganciclovir failure as prophylaxis in SOT are adverse effects and the development of resistant mutations (20, 21). In our observation, we described two cases of LMV secondary prophylaxis. In the first case, the patient developed a valganciclovir/ganciclovir resistant mutation (L595S) that compelled a switch to LMV; in the second case, neutropenia caused by valganciclovir/ganciclovir precipitated the use of LMV.

These two cases together with the reviewed bibliography suggest that alternatives to valganciclovir are needed. Even though LMV seems to be a potential drug for prophylaxis regimens, it should be used with caution because of the early development of resistance. This was exemplified in the second case of our study, in which LMV administration caused C325F-resistant mutation to emerge in *UL56*. It has been suggested that the *UL56* LMV target gene has a low genetic barrier to mutate under antiviral selection pressure (22) but not without (10); therefore, exposure to low doses of LMV may lead to the development of *UL56* mutants (23). This study together with previous trials showed that LMV resistance is frequent when used as secondary prophylaxis, which could be caused by the continuation of LMV therapy even under CMV relapses. Thus, it has been highly recommended to initiate LMV secondary prophylaxis when CMV loads are undetectable in three or more consecutive tests to avoid *UL56* resistance development. Clinical experience showed that primary prophylaxis with LMV does neither prevent CMV blips, which should not imply a risk of resistance if dosage is correct and there is not treatment interruption (1, 2).

Most of the literature about LMV secondary prophylaxis refers to clinical cases in which LMV was associated with nonresponse or the development of antiviral resistance, which bias the clinical use of LMV for the mentioned indication. Therefore, larger clinical trials and well-designed observational cohort studies are needed, which can only be achieved through collaborations or multicenter groups.
